# Women’s Experiences Caring for Their Husbands’ Siblings With Developmental Disabilities

**DOI:** 10.1177/2333393615604169

**Published:** 2015-09-06

**Authors:** Yeh-chen Kuo

**Affiliations:** 1 National Taipei University of Education, Taipei City, Taiwan, R.O.C

**Keywords:** caregivers, Chinese culture, sister-in-law, sibling, developmental disabilities

## Abstract

A phenomenological method was used in this study to examine the experiences of women caring for the husband’s sibling with developmental disabilities (DDs) with the aim of establishing how and why they came to care and continued to care for them. Three *themes emerged* after drawing on stories shared by seven women: for the sake of my husband, powerlessness, and trade-off between cost and rewards. The findings of this study show that Taiwanese women accept the cultural norms, thus accepting the caregiving responsibility. Reciprocity did not help determine whether women started caring for the husband’s sibling with DD. However, when an imbalance in reciprocity is present, women experience negative emotions that often result in tension within the family. Positive factors contributed by the husband and parents-in-law can facilitate the work of caregivers by ameliorating physical pain and psychological distress that can occur during the caregiving process.

The increased life expectancy of persons with developmental disabilities (DDs; for example, intellectual disability [ID], cerebral palsy, or Down’s syndrome) presents challenges not only for the government but also for the very fabric of family life. Many of these people will become frail and in need of physical, social, and emotional support. For example, people with Down’s syndrome commonly suffer a substantial deterioration in their ability to perform activities of daily living (ADL) after the age of 40 years (Maaskant et al., 1996), and those with cerebral palsy are exposed to more medical problems and greater decrements in ADL functions with age compared with their counterparts with ID of no specific etiology (Lifshitz, Merrick, & Morad, 2008).

Separate residences for aging family members and those with disabilities are still not a dominant pattern of residence in Taiwan; for example, 92% to 95% of people with ID live with their families (Chou & Schalock, 2007). When mothers and/or fathers who have been lifetime providers of care to people with DD are no longer able to fulfill this role, the most likely substitute caregiver is a sibling. The aim of this study was to characterize experiences of women caring for the husband’s sibling with DD at home. The rationale underlying the focus on these women is informed by Confucianism, a dominant moral philosophy that guides the culture and behavior of people in Taiwanese society (Cai, 2004).

In the Analects (Confucius, 1998), the notion of *Nei-Wai* has been applied to the conceptualization of gender, which addresses the assignment of functions through division of labor: Women handle the domestic affairs, such as nurturing children, cooking, weaving, and other household work, whereas men handle public and social affairs, such as farming, commerce, and literary learning (Chan, 2000). Based on the important roles that women have always played in the traditional Chinese family, it is plausible that they will also be called upon to care for family dependants of their husbands, such as their siblings with DD (Chao & Roth, 2000; Kao & McHugh, 2004).

Two main concerns led to this study. First, like many other recently developed nations in the East, contemporary Taiwan society is blending traditional Confucian culture and Western culture by assimilation of Western values related to family structure and appropriate gender roles (H. C. Hsu & Shyu, 2003; Tong, 2007). Moreover, women belong to multiple social groups, which may result in exposure to changing sets of norms over time. The varied experiences that women have throughout life may affect their understanding of caregiving obligations (Lee, 2010). To the best of my knowledge, no previous empirical study has explored why women provide care to the husband’s sibling with DD in Taiwanese society. To broaden the knowledge base and provide a comprehensive understanding of women’s caregiving, it is important to ask why—given a lack of previous bonding and blood ties—women end up caring for the husband’s sibling with DD.

The second concern that led to this study relates to the influence of the hierarchy in a traditional Chinese family on the experiences of women caring for the husband’s sibling with DD. To be specific, the Confucian ideology of *Shan-Shia* (“superior-subordinate”) dictates how family members ought to behave toward each other in an ordered hierarchy and that inferiors are expected to be subordinate to their superiors (F.-K. Hsu, 1975; Hwang, 1999; Nishi et al., 2010). Within this ordered hierarchy, culturally induced beliefs dictate that a woman should follow the lead of her husband and prioritizes the benefits to the husband (Chen, 2003).

Patrilocality plays a role in setting binding constraints to obligate women to follow the hierarchical order, by placing them in a subordinate position in the family. Patrilocality involves a couple residing at the husband’s home, so that upon her marriage, the wife transfers her obligation from her family of origin to her husband’s family (Gupta et al., 2003). As a newcomer compared with others in her husband’s family, the woman is thus always an object of deep suspicion and her position in the family is subordinate not only to all of the men but also to the more-senior women (Gupta et al., 2003). To the best of my knowledge, there is no empirical evidence regarding the implications of such cultural influences and practice upon the experiences of women caring for the husband’s sibling with DD.

## Theoretical Framework

Reciprocity is important for finding satisfaction and meaning in the caregiving relationship (Cartwright, Archbold, Stewart, & Limandri, 1992). This concept has most often been studied from the perspective of equity theory (Walster, Walster, & Berscheid, 1978). Based on the possibility that the presence of satisfaction and meaning in the caregiving relationship might contribute to a woman’s willingness to continue to care, equity theory was chosen as a theoretical framework for the present study, to identify the reciprocity and exchange relationships in the caregiving process. According to equity theory, individuals have a tendency to pursue reciprocity in interpersonal relationships, and those who find themselves participating in an unreciprocated relationship will become distressed (Schaufeli & Buunk, 2003). The greater the inequity or lack of reciprocity that exists, the more distress individuals will experience (Walster et al., 1978).

Many studies have shown that perceptions of inequity are associated with negative emotion among couples dealing with chronic illness (Kuijer, Buunk, & Ybema, 2001; McPherson, Wilson, Chyurlia, & Leclerc, 2010; Ybema, Kuijer, Buunk, De Jong, & Sanderman, 2001; Ybema, Kuijer, Hagedoorn, & Buunk, 2002). For example, Ybema et al. (2002) assessed caregivers’ burnout among intimate partners of patients with a severe illness, and found that intimate partners of both cancer patients and patients with multiple sclerosis were relatively more likely to experience burnout when they felt that they did not benefit sufficiently in their exchanges with their ill partner. Higher perceptions of inequity were strongly associated with higher emotional exhaustion.

Given the association between caregivers’ perceptions of inequity and negative emotion, several studies have attempted to identify the exchange factors that contribute to caregivers’ emotional well-being, decrease their burden, and improve family relationships. One of these factors is intrinsic rewards. For example, Carruth, Tate, Moffett, and Hill (1997) tested a theoretical model developed to explain family satisfaction among 171 caregivers of elderly parents. They found that family satisfaction was both directly and indirectly influenced by reciprocity, in that intrinsic rewards derived from giving care contributed to higher levels of family satisfaction. Similarly, daughters-in-law were more likely to experience the costs of caregiving without potentially compensatory resources (Ingersoll-Dayton, Starrels, & Dowler, 1996).

Besides material tangible support, there is evidence that exchanges of a more interpersonal nature such as love, warmth, and affection may still be important for their caregivers’ well-being (McPherson, Wilson, Chyurlia, & Leclerc, 2011; Reid, Moss, & Hyman, 2005). For example, Reid et al. (2005) assessed whether self-esteem and intrinsic motivation influence the relationship between reciprocity and caregiver burden for caregivers of frail older adults with illness or disabilities, and found that exchanges of respect, regard, and commitment between caregivers and care recipients were associated with lower levels of developmental, physical, social, and emotional caregiver burden. Conversely, an analysis of 140 caregivers of elderly relatives showed that for the daughters-in-law who were considered to be the main caregivers, more negative appraisals of the caregiving were associated with less positive affect (Pruchno, Peters, & Burant, 1995).

In a Taiwanese context, some unique, implicit cultural exchanges were found by H. C. Hsu and Shyu (2003), who used in-depth interviews with inductive analysis to develop a conceptual framework for exploring social exchanges and their implicit calculations for caregivers in Taiwan. It was found that in the process of caregiving, implicit exchanges such as rewards from public opinion might be intermediary factors in helping caregivers to cope with their burden, or may even influence their continuation of care.

Most of the studies in the literature have focused on couples dealing with chronic illness in a spouse, or on women giving care to their parents-in-law. It is not yet clear how women’s perceptions regarding equity in the caregiving relationships influence their decision to continue that care. To address this gap in the existing literature, the aim of the present study was to answer these questions based on equity theory.

The research questions leading this study were as follows:

**Research Question 1:** What are the experiences of women caring for the husband’s sibling with DD?
**Research Question 2:** Given the lack of prior bonding and blood ties, how and why do these women end up caring for and continuing to care for their husband’s siblings with DD?

## Research Method

This study explored the experiences of women caring for the husband’s sibling with DD with the aim of establishing how and why they came to care and continued to care for them. A phenomenological approach was used to explore the day-to-day lived experiences of women who take care of the husband’s sibling with DD at home. The intention was to uncover the essence of, describe, and interpret the meaning of their day-to-day lived experiences, and then to determine the patterns of these women’s caregiving experiences.

Data collection in heuristic research begins with self-reflection. Heidegger (1962) proposed that the experience of *Dasein* (“being there”) was the starting point for the hermeneutic method. Being is reportedly first experienced through what he termed the “fore structure of understanding,” and then expands through a preliminary grasp of the structures of being to a comprehension of being itself. The “fore structure” can be used to understand the existential and renewed experience of Dasein (Penoyer, 2005). Therefore, based on my own background and experience, I attempted to understand the experiences of women caring for the husband’s sibling with DD by establishing how and why they came to care for and continued to care for them. To implement this important principle of phenomenological philosophy, I engaged in self-reflection by taking notes during each phase of the research process, and was careful not to impose my points of view when interpreting the experiences reported by the participants.

## Research Design

### Sampling

Participants for this study were recruited using purposeful and snowball sampling (Patton, 1990), selecting those who had some depth of experience with the study questions and who were willing and able to reflect upon those experiences. The criteria for choosing research participants had to match with the questions and purpose of the inquiry; the inclusion criteria were women (a) who had a husband with a sibling with DD, (b) who lived with that sibling, and (c) who self-identified as being the one most involved in the caregiving to that sibling with DD at the time of being interviewed. Two types of recruitment technique were used: (a) an advertisement posted in the newsletters and bulletins of universities, social-service agencies, institutions, self-help groups, and organizations that provide services to people with DD and their families and (b) snowball sampling. This strategy has been shown to work well for identifying members of a population who are generally hard to reach (Rubin & Babbie, 1997).

Morse (1994, 2000) recommended that phenomenological studies aimed at identifying the essence of experiences should typically include 6 to 10 participants, and considered that the number of participants required in a specific study to reach saturation was influenced by the amount of useful information that could be obtained from each participant. The phase of data collection for the present study was completed when seven participants had been recruited, since then sufficient useful information had been obtained from each participant to determine themes and categories, and no new information was forthcoming to construct representations of the phenomenon and its variability (Byrne, 2001). Three of the participants were recruited through social-service agencies and three by snowball sampling; all of them were married. The highest level of education reached by the participants was high school (*n* = 4) or college (*n* = 3), and their age range was 28 to 63 years (42.40 ± 7.25 years, *M* ± *SD*). The age range of the siblings with DD was 25 to 55 years (35.14 ± 8.12 years).

#### Data collection

Interviews were conducted between August and December 2013 after permission was obtained from the Research Ethics Committee, National Taiwan University. Participants who indicated an interest in the study were contacted to confirm that interest and to inform them of the study parameters; they were told of the purpose of this study, how the findings would be used, and what their involvement entailed. They were informed that they would be interviewed twice and that additional contact might be requested to clarify the content and interpretation of their narratives. The interviews were to be audio-recorded and their experiences would be kept confidential by using randomly assigned pseudonyms in each phase of the research process and publication. Before the interviews began, the possibility that discussing how they experienced their role as a sister-in-law of a person with DD might cause some uncomfortable emotions and thoughts was discussed with each of the participants. Contact information for counseling agencies was provided in case that situation arose. The participants were assured that they could withdraw from the study at any time without any consequence. This information was clearly stated in the informed consent form.

Mutually convenient times for face-to-face interviews were arranged with the participants. The interview locations were chosen by the participants according to where they felt a meeting would be most comfortable for them. An information letter and two copies of the consent form (one that would be kept by the participants and the other by the researcher) were presented to participants before the interviews began.

Data were collected through in-depth, semistructured interviews, which were designed so that the researcher could first introduce the topic and then guide the discussion by asking specific questions (Rubin & Babbie, 1997). The first set of questions of the interview guide sought to reveal the caregiving experiences of the women in relation to the husband’s sibling with DD. Typical questions asked in this set included “What sort of assistance do you provide to your husband’s sibling with DD?” and “How would you describe your caregiving experiences?” Another set of questions was designed to gather information about how and why they came to care and continued to care for that sibling with DD. Questions asked in this set included “What made you become the caregiver of your husband’s sibling with DD?” “Were you asked to take care of your husband’s sibling, or did you volunteer without being asked?” “During the time when you were the caregiver to your husband’s sibling with DD, what kinds of give-and-take occurred in this caregiving relationship?” and “Why do you continue to take on this responsibility?” The duration ranges for the first and second interviews were 93 to 121 and 66 to 87 minutes, respectively. The study participants were contacted via telephone or email when necessary after reviewing of the transcribed interviews in an effort to clarify any confusing data. Detailed notes were taken of any such conversation or correspondence that took place, which were incorporated, together with any associated insights, into the data analysis.

#### Data analysis

The materials used in the analysis included transcripts of the participants’ narratives from interviews, my own notes and analytic memos, and telephone or email communications if applicable. The materials, software, and procedures used in the data analysis process are detailed in this section.

All interviews were audio-recorded and then transcribed verbatim by research assistants into written descriptions of the participants’ lived experiences (Van Manen, 1990). The second interviews with the participants were conducted after the previous one had been transcribed and analyzed. The data were analyzed at the time of transcription and between interviews to ascertain that there were sufficient data to represent the phenomenon and its variability. This concurrent and simultaneous method allowed for critical examination and use of previous experiences, and was crucial in determining the completeness of the data. When thematic analysis and determination of themes were complete, they capture the essence of the phenomenon (Swanson-Kaufman & Schonwald, 1988).

Multiple analytic memos (Maxwell, 1996) were written after each interview, as well as throughout the analysis process, allowing me to record and begin to categorize my initial and ongoing reflections, questions, ideas, and analytic decisions. In addition, telephone or email communications with participants were used to derive meanings and for interpretation if applicable.

NVivo 9 qualitative data analysis software (QSR International, 2011) was used to encode the data. This software enables a researcher not only to manage a huge volume of textual documents but also to store and retrieve data, and it helped with the coding process. A modification of the Stevick–Colaizzi–Keen method (Colaizzi, 1978) was used in the analysis, which has been used frequently in other phenomenological studies.

In the first stage of coding process, words, phrases, and sentences that represented essential elements of experiences of women caring for the husband’s sibling with DD were identified with the aim of establishing how and why they came to care for and continued to care for the sibling (Van Manen, 1990). This process initially generated 30 codes from 682 passages in the data. Examples of initial subthemes emerged from these codes include “responsibility to my husband” and “benefits to the husband come first.” This was followed by developing a framework of major themes within the subthemes. The initial subthemes of “responsibility to my husband” and “benefits to the husband come first” were combined into the major theme of “for the sake of my husband.” These emerging themes provided insights into how these women made sense of the events and situations in their lives. In the final stage, I constructed an overall description of the meaning and essence of the experience of caring for the husband’s sibling with DD with the aim of establishing how and why they came to care for and continued to care for them.

### Ensuring Rigor

Criteria for trustworthiness are often used to judge whether the quality and extent of study findings are believable to readers (Schwandt, 1997). The assessment of trustworthiness of the present study was based on the dimensions of credibility and transferability (Lincoln & Guba, 1985). The procedures for achieving all of these criteria are discussed explicitly in this section.

#### Credibility

In qualitative inquiry, credibility is the extent to which the findings accurately reflect the views of the participants (Lincoln & Guba, 1985). The design of the present study contained three strategies for ensuring credibility: (a) reflection, (b) member checking, and (c) peer debriefing.

##### Reflection

In heuristic research, data collection begins with self-searching. As Schutz (1967) stated, “Everything I know about your conscious life was really based on my knowledge of my own lived experiences” (p. 106). The present study sought the meanings that the experience of being a sister-in-law of a person with DD had for the participants. This understanding was reached from my own viewpoint of being a social worker who has worked with persons with DD in hospitals and a self-help group (i.e., historical situations within which my understanding took place). However, in attempting to understand my participants’ interview texts from my particular point of view did not mean I blindly imposed my point of view on their texts. During data analysis, I employed reflective journaling to record aspects of the interview and personal reflections. In doing so, I was drawn into conversation with my own historical texts as well as those of the participants. Conversing with the texts was an opportunity for me to compare my own history as a daughter-in-law with those of the participants, which contributed to the trustworthiness and provided an audit trail for this study (Smith, 1999). For example, during the interviews it was evident that all of the participants exhibited strong negative emotions when they described their experiences of having disagreements with a family member of their husband; this was recorded as “why do they have such a strong reaction?” in the notes. In the phase of data analysis, I read the sentences written in my notes and recalled that as a daughter-in-law myself, the opinions of my husband’s family were always prioritized over my own opinions. I thus speculated that their strong negative reactions to the disagreement might be because of their feeling of powerlessness due to them not being able make any decisions regarding whom they were caring for. This speculation was supported by all of the participants in the member-checking phase of the study.

##### Member checking

Member checking, which refers to seeking feedback from study participants about the data extracted from their respective stories (Guba & Lincoln, 1989), ensures the mutual believability between the researcher and the participants regarding the data (Lincoln & Guba, 1985). Miles and Huberman (1994) suggested that this feedback can be done after the data analysis has been completed. The seven participants in the present study were invited to read and react to the findings obtained, and all of them provided feedback to the researcher via telephone. Lisa responded that the theme of powerless captured the reason why her opinion was not considered when she advised her parents-in-law not to buy a mail-order bride for her brother-in-law with ID. The rest of the participants responded to the presented findings with “yes, the findings do reflect my experiences.” Their reflections indicated that participants felt that the findings fully represented the essence of their experiences.

##### Peer debriefing

Peer debriefing involves the use of an outside expert to check on the inquiry process. These colleagues must be peers of the researcher and have a solid understanding of the methodological issues involved in the research (Lincoln & Guba, 1985). In this stage, peer debriefing was achieved through ongoing discussion with a professor who had expert knowledge about the characteristics of Taiwanese culture, which involved sharing ideas and posing and answering questions about the methods and procedures used in study, and the interpretation of the data.

#### Transferability

Transferability refers to the extent to which study findings can be generalized to other settings. The use of thick description in the presentation of the findings will contribute to transferability. The detailed description of the experiences of the participants and the context of the data collection assist readers in judging the extent to which the findings can apply to other individuals in other settings (Lincoln & Guba, 1985). Malterud (2001) argued that although the aim of research is to produce information that can be shared and applied beyond the study setting, no study can provide findings that are universally transferable. Based on this argument, I formulated the criteria used for choosing research participants (see “Sampling” section); these criteria thoroughly considered what an adequate degree of transferability would be.

## Findings

The participants’ responses provided unique insights into the experiences of women caring for the husband’s sibling with DD and, given the lack of prior bonding and blood ties, how and why they came to care for them. Three main themes emerged: (a) for the sake of my husband, (b) powerlessness, and (c) trade-off between cost and rewards.

### For the Sake of My Husband

This was the theme used to explain why the women came to care for the husband’s sibling with DD despite the lack of previous bonding and blood ties. The predominant reason given in response to the question “What made you become the caregiver of your husband’s sibling with DD?” was cultural belief rooted in the Confucian ideologies. One of the participants stated, “The mission of a woman is to support her husband, which allows him to put all his effort on developing his career with no worry about trivial things that happen in the family.” Her response reflected the Confucian ideology of Nei-Wai (“in-out”), which dictates that women should handle the domestic affairs whereas men should handle public and social affairs (Chan, 2000; Confucius, 1998).

Some of the responses reflected the Shan-Shia ideology dictating that women should follow the lead of their husband. One of the participants was assured by her husband before they married that she would not be responsible for taking care of her brother-in-law with ID in the future. However, that promise was not kept and she eventually became the main caregiver to her brother-in-law when her mother-in-law spent more time with her father-in-law abroad for business:

She [her mother-in-law] changed her mind and decided my brother-in-law is our responsibility. My husband did not oppose what she said. What else can I do? It is not like I can say [to my husband] you promised me [that I don’t need to take care of him]. If you marry a chicken you follow the chicken, if you marry a dog you follow the dog.

When I asked “During the time when you were the caregiver to your husband’s sibling with DD, what kinds of give-and-take occurred in your caregiving relationship?,” most of the participants responded that they did not take reciprocity into consideration. Their responses reflected the Shan-Shia ideology that women are supposed to prioritize the benefits to their husbands, even when performing as a caregiver was to their own detriment (Chen, 2003; Confucius, 1998; F.-K. Hsu, 1975):

He [the husband] worked here [the family business]. If I decline the arrangement [to be a caregiver] and argue as to why my elder sister-in-law was spared from the caregiving work, or find a job outside the family, it would put my husband in a very difficult position.

I could not be selfish. Everyone has his/her share of responsibility in this [extended] family . . . My sister-in-law does the accounting and I do the caregiving.

In sum, reciprocity was not one of the factors determining whether women started caring for the husband’s sibling with DD. Instead, their decision and motivations were influenced by the Nei-Wai and Shan-Shia ideologies (Chan, 2000; Chen, 2003; Confucius, 1998; F.-K. Hsu, 1975; Hwang, 1999; Nishi et al., 2010).

### Powerlessness

The powerlessness theme was used to describe the implications of cultural influences and practice upon the experiences of women caring for the husband’s sibling with DD. Some of the participants in this study tried to promote the physical and psychological well-being of the husband’s sibling with DD by training them to behave well and to have a healthy lifestyle, or providing them with technological care. However, due to their position in the family as a newcomer compared with other members of their husband’s family, when participants had different opinions, tensions in the family arose and the opinions of their husband’s family members were always prioritized over that of the participants. The participants received no respect for their opinions in return for their caregiving, and so experienced frustration and dissatisfaction in the caregiving relationships:

His obesity has become a big problem to his health . . . To encourage him to exercise more, I made a deal with him: “if you run three circles of the playground, then I will buy ice cream for you. If you don’t, then you will have to watch us eat it.” He played dumb and sat still beside the playground. To show him I meant it, I bought ice cream for my parents-in-law and myself. He turned to my mother-in-law and asked her to give him some. I asked my mother-in-law do not indulge him, my mother made a long face [a facial expression to show unpleasantness] to me and said: “He is bitter enough that I gave him such a crippled body, now you want me to deprive his limited pleasure. Give him a break!” I felt frustrated that every time I try to establish a rule that is good for my brother-in-law, my mother-in-law sabotages it . . . what can I do? She is the senior in this family.

It is wonderful if he can talk to us directly [via an eye-tracking device]. I told my mother-in-law the idea, for some reason, she did not approve it . . . she said the only healthy part he has left is his eyes. The TV [screen] was too small, which would hurt his eyes. The answer was so weird. Later my husband’s sister told me the real reason. To make him gain the ability to speak, my mother-in-law visited a psychic in a temple and asked him to negotiate with the ghosts [she believed] who were haunting him. The psychic told her: Your son was not haunted by ghosts. Your son has a special gift. He can see things [people’s future]. The higher spirit shut his mouth to prevent him from leaking the destinies of people. Once he gains the ability to speak, there would be a consequence for him. This is ridiculous! But I had better shut my mouth. If [I insisted to have him use the eye-tracking device and] something happens to him, I will be blamed.

Very often, the participants in this study were powerless and felt that they were misunderstood when they proposed an idea based on goodwill, which had been suspected as having the intention to offload the caregiving responsibility (Gupta et al., 2003). One of the participants shared her opinion with her mother-in-law that having a job, even as a janitor, would be a wonderful thing for her sister-in-law with ID. She described her mother-in-law’s reaction as “she was so convinced that I was planning to abandon my husband’s sister step by step. The first step is to ask her to work.”

They experienced negative emotions when they received no positive feedback for their goodwill. One of the participants had a brother-in-law with DD who was obsessed with a certain kind of slipper. Almost every time he went to the night market, he had to buy a pair:

I told my husband to tell his brother not to waste money. My stupid husband lost his temper and hit him hard. My mother-in-law said that I am a manipulator who tried to create sibling rivalry and lead my husband to abandon his brother. I felt very sad about what she said. What I did was for his own good.

Another participant felt she was misunderstood as intending to take advantage of her brother-in-law with ID when she tried to stop her parents-in-law from buying a mail-order bride for him:

The broker said the mail-order bride will “take care of him for the rest of his life.” I did not agree with him. When my parents-in-law were browsing the photos of candidates, I told them it was probably not a good idea. How could we make sure he would be well treated under the circumstance of no love as a foundation. The mail-order bride will probably run away when she got her status as a permanent resident . . . Or worse, take the property which is under my brother-in-law’s name. To be honest, what I did not say is, it is not fair to that woman. My parents-in-law did not respond to my concerns. A few days later I went to the barber shop to have my hair done. A very good friend of my mother-in-law advised me not to talk too much. She said my mother-in-law told her the reason I tried so hard to against the idea [of buying a mail-order bride] was because if my brother-in-law got married, then I will not be able to control the share of my brother-in-law’s money in the future. I felt insulted and just let them do whatever they wanted.

This theme illustrated the powerlessness of participants in the decision-making process. They received no respect or positive feedback for their opinions or goodwill, or even experienced negative emotions in the caregiving process, especially when they were suspected by their parents-in-law of trying to offload the caregiving responsibility or of taking advantage of the husband’s sibling with DD.

### Trade-Off Between Cost and Rewards

The theme of the trade-off between cost and rewards was used to describe how women balanced the costs and rewards associated with caring for the husband’s sibling with DD, which is important for maintaining caregiving relationships. Despite the emotional and/or physical cost to these women in caring for the husband’s sibling with DD, the participants mentioned several factors that motivated them to continue to be the caregiver.

The participants in this study experienced physical pain and psychological distress such as fatigue, frustration, anxiety, and worry in the caregiving process. One of the participants had been taking care of her brother-in-law with ID for more than 20 years. She has been constantly worried what will happen if her brother-in-law outlives her:

My sister has invited me to travel abroad with her several times and I have always declined her invitation. It costs money. I want to save money in case I am gone before him [her brother-in-law with ID] and my children would not take care of him. I know my children would [take care of him after I am gone]. However, I am not sure their spouses would. So I need to save the money as much as possible in case something happens to me.

Another participant had been constantly worried because the sister with ID disappeared all the time:

She sneaks out whenever she can. I try my best to keep an eye on her, but she disappears all the time. There was one time when she was missing for more than 10 hours. I knelt down and prayed to Buddha—please help us to find her. She has been like this since before I married into this family. They know it is not easy to keep her at home. However, if she disappears forever, my husband and his parents will never forgive me. Keeping an eye on her and trying not to lose her makes me very nervous.

The psychological distress was sometimes exacerbated, especially when their efforts in providing care were not explicitly appreciated by other family members:

Do you know how much strength I have to use to lift her? Do you know how difficult to communicate with a foreign helper when she does not understand what I want her to do? Does anyone really understand my fatigue, my physical pain, and my frustration? . . . My husband’s sister said I am lucky because I don’t need to work. I felt I was deeply misunderstood and feel upset about this kind of comment. It is not like I do nothing at home. She [the foreign helper] shares only part of the work. I still need to do the lifting and bathing work and am bound to this house with her. It is not fair to say so.

Another participant repeatedly mentioned that she was constrained to staying at home and had no social life because of her caregiving role. This again illustrated that social isolation can be exacerbated by not receiving any appreciation in return from other family members:

My husband’s two brothers did not want to rotate [the caregiving responsibilities] with us. So they pay me [according to the market price] instead . . . I hate the feeling they treat me like a maid . . . For example, there was one time I had a reunion, so I asked my two sisters-in-law to keep an eye on her. They were reluctant to do so. I was not happy and complained that I have lost my contact with friends [because of being a caregiver] for this family. One of my sister-in-laws blurted out, “we already pay you!” It was so humiliating. They treat me like a maid . . . like paying me money is enough.

Given the emotional and/or physical cost to these participants of caring for the husband’s sibling with DD, what had motivated them to continue to be the caregiver? The participants mentioned several motivating factors. The first was a sense of pride. One of the participants took over the caregiver’s responsibility from her mother-in-law more than 20 years ago. She was proud of herself because “I make sure our dining table always has a place for his chopsticks and bowl. I treat him no different from my own children.”

The second factor is their bonding with the sibling with DD. One of the participants described her relationship with her brother-in-law with ID as “even closer than with my children” and “he is the one who is always beside me and follows me everywhere.” The answers of another participant mixed the sense of pride and her bonding to her brother-in-law with ID:

In the past, my mother-in-law indulged him. Whenever there was none of his favorite food on the table, he swept all the plates to the ground. I did not like him at all . . . Since my mother-in-law stayed abroad for longer times with my father-in-law, I have more authority with my brother-in-law [with ID]. With the consent of my husband, I registered him [the brother-in-law with ID] for a day program. The teacher has taught him well. He has become cooperative and very close to me. He has told me secrets and asked me not to tell his brother; he politely asked me to cook what he likes; he was very happy when I took him bicycling; I shed tears after I saw his performance in the music band . . . He is such an angel. It is nice to have him.

The third factor motivating the participants to continue to care for the husband’s sibling with DD was the desire to do something in return for what they had received from their husband and parents-in-law. One of the participants explained that she would continue to be a caregiver because whenever her mother-in-law complained about her, her husband asked the mother to say no more. She described her husband as “conscientious” and “supportive.” Two participants expressed their gratitude for the support received from their parents-in-law in various forms, such as intrinsic rewards, public expressions of appreciation, and other acknowledgements of their contributions, which enabled them to continue to be caregivers:

Why wouldn’t I want to take care of him? They have been so nice to me. My parents-in-law praised me in front of my neighbors and relatives of my own family . . . They have an apartment in the city. Every month after I collect the rent and give it to my parents-in-law, they put some in their pocket and give the rest of the money [approximately half the market price for a formal caregiver] to me.

My husband is a business man. He used to turn off his cell phone and come home late all of the time. There was a period of time when the situation got worse and became intolerable. I went back to stay with my own family. My parents-in-law sent my elder sister-in-law to deliver a message to me. They had given a serious warning to my husband not to go too far and asked him to cherish what I have done for his family. I went home right away. They know what I have done for this family and stand on my side when their son has gone too far.

In this theme, women provided much of the care required by the husband’s sibling with DD and as a consequence experienced physical pain and psychological distress. However, positive factors such as a sense of pride and the bonding with the sibling with DD, and the desire to do something in return for what they had received from their husband and parents-in-law enabled them to continue being caregivers. Determining the trade-off between costs and rewards therefore seems important for the continuation of caregiving relationships.

## Discussion

The lived experiences of seven women who care for the husband’s sibling with DD despite not having prior bonding and blood ties were examined in this study, and the reasons how and why they came to become and continued to be the caregiver were established. The findings of this study indicate that the decision and motivations for women to be caregivers to the husband’s sibling with DD are still influenced by traditional Confucian culture. They did not take reciprocity into account when they made the initial decision to provide care. The participants’ accounts revealed that the Confucian ideology of Shan-Shia dictates that women should put the needs and interests of their husbands ahead of their own, even if serving those needs and interests would be at the expense of their own (Chen, 2003; Confucius, 1998). For example, one of the participants stated that if she declined the arrangement “it would put my husband in a very difficult position.” Moreover, the Confucian conception of Nei-Wai tradition, which allocates caregiving tasks to women, still has a profound effect on decisions by women to take on a caregiver’s role (Confucius, 1998). In summary, the findings of this study indicate that patriarchy beliefs and cultural norms still deeply influence the decision of Taiwanese women to provide care even in the absence of reciprocity in the caregiving relationships.

As some of the participants were reluctant to take on the role of caregiver for the husband’s sibling with DD but still did so, the cultural ideology underpinning that decision must be challenged. This cultural belief system overlooks, neglects, and ignores the needs and desires of women, and operates for the benefit of the men and to ensure optimal functioning of the patriarchal system (Kuo & Lach, 2012). Encouraging the family to consider other possibilities such as the men staying at home to provide care, or seeking an independent living program for the persons with DD, would allow women to attend to more of their own needs and enable them to make a considered decision to care or not to care.

Consistent with the argument of Gupta et al. (2003), the participants in the present study were powerless in the decision-making process regarding whom they were caring for due to their subordinate position in the family. Because they received no respect or positive feedback for their opinions or goodwill, they experienced negative emotions, especially when they were suspected by their parents-in-law of intending to try to offload the caregiving responsibility or to take advantage of the husband’s sibling with DD (Kuijer et al., 2001; McPherson et al., 2010; Ybema et al., 2001; Ybema et al., 2002). This finding reveals that although reciprocity was not one of the determinants for women ending up caring for the husband’s sibling with DD, the presence of an ongoing imbalance in reciprocity can induce negative emotions that often result in tension within the family (Schaufeli & Buunk, 2003; Walster et al., 1978).

To reduce the degree of tension within the family, services should be provided to improve communication, decision-making, and conflict-management skills when they encounter problems or have disagreements regarding the issues of caregiving methods or placement decisions (Nosek et al., 2004), especially when the women’s voices are metaphorically muted due to their subordinate position in their husband’s family.

Moreover, some of the tensions arose from parents-in-law worrying about the future of their offspring with DD. To ease that worry, the Taiwanese government should focus on developing appropriate long-term care policies to ensure that these offspring will be well taken care of when their parents or families are no longer able to provide such care. The implementation of a comprehensive system of services based on such care policies could also provide a solid foundation with which to modify the ideology underlying the cultural norm and patriarchy beliefs that require women to provide care in Taiwanese society.

In the theme of the trade-off between cost and rewards, women provide much of the care required by the husband’s sibling with DD, which can result in them experiencing physical pain and psychological distress. However, positive factors such as a sense of pride and the bonding with the sibling with DD, and the desire to do something in return for what they had received from their husband and parents-in-law such as intrinsic rewards, public expressions of appreciation, and other acknowledgements of their contributions enable caregivers to continue to provide care. The findings of this study support the theoretical construct of equity and are consistent with previous findings that the trade-off in costs and rewards is important for the continuation of caregiving relationships (Carruth et al., 1997; H. C. Hsu & Shyu, 2003; Reid et al., 2005; Walster et al., 1978; Ybema et al., 2002). I therefore recommend the implementation of professional interventions that will help family members to enhance their ability to maintain reciprocity in the caregiving relationship, and especially to help families to acknowledge and express their appreciation for the contributions made by women. The finding of this study that psychological distress can be exacerbated when the efforts made by women in providing care are not explicitly appreciated by other family members indicates the paramount importance of expressing appreciation to caregivers.

It is worth noting that these services need to be used with caution within the Taiwanese cultural context, which emphasizes “face-saving.” The aforementioned services might intrude into the family’s desire to keep their business private within the family, thus making them reluctant to seek help. As such, practitioners may need to focus on concrete benefits that the families can garner from receiving such help to counterbalance the possible hesitation that they may experience (Kuo & Geraci, 2012).

Further exploration of the experiences of women caring for the husband’s sibling with DD with the aim of establishing how and why they started caring for and continued to care for them in other cultures would enhance our understanding of caregiving.
